# Risk analysis of pulmonary metastasis of chondrosarcoma by establishing and validating a new clinical prediction model: a clinical study based on SEER database

**DOI:** 10.1186/s12891-021-04414-2

**Published:** 2021-06-09

**Authors:** Wenle Li, Shengtao Dong, Haosheng Wang, Rilige Wu, Huitao Wu, Zhi-Ri Tang, Junyan Zhang, Zhaohui Hu, Chengliang Yin

**Affiliations:** 1 Department of Orthopedics, Xianyang Central Hospital, Xianyang, 712000 China; 2 Clinical Medical Research Center, Xianyang Central Hospital, Xianyang, 712000 China; 3 Department of Spine Surgery, Second Affiliated Hospital of Dalian Medical University, Dalian, 116000 China; 4 Orthopaedic Medical Center, The Second Hospital of Jilin University, Changchun, 130000 China; 5 Medical Big Data Research Center, Medical Innovation Research Division of Chinese PLA General Hospital, Beijing, 100853 China; 6 National Engineering Laboratory for Medical Big Data Application Technology, Chinese PLA General Hospital, Beijing, 100853 China; 7 Intelligent Healthcare Team, Baidu Inc., Beijing, 100089 China; 8 School of Physics and Technology, Wuhan University, Wuhan, 430072 China; 9 Department of Spine Surgery, Liuzhou People’s Hospital, Liuzhou, 545000 China; 10 Faculty of Medicine, Macau University of Science and Technology, Macau, 999078 China

**Keywords:** Nomogram, SEER, Chondrosarcoma, Lung metastasis

## Abstract

**Background:**

The prognosis of lung metastasis (LM) in patients with chondrosarcoma was poor. The aim of this study was to construct a prognostic nomogram to predict the risk of LM, which was imperative and helpful for clinical diagnosis and treatment.

**Methods:**

Data of all chondrosarcoma patients diagnosed between 2010 and 2016 was queried from the Surveillance, Epidemiology, and End Results (SEER) database. In this retrospective study, a total of 944 patients were enrolled and randomly splitting into training sets (*n* = 644) and validation cohorts(*n* = 280) at a ratio of 7:3. Univariate and multivariable logistic regression analyses were performed to identify the prognostic nomogram. The predictive ability of the nomogram model was assessed by calibration plots and receiver operating characteristics (ROCs) curve, while decision curve analysis (DCA) and clinical impact curve (CIC) were applied to measure predictive accuracy and clinical practice. Moreover, the nomogram was validated by the internal cohort.

**Results:**

Five independent risk factors including age, sex, marital, tumor size, and lymph node involvement were identified by univariate and multivariable logistic regression. Calibration plots indicated great discrimination power of nomogram, while DCA and CIC presented that the nomogram had great clinical utility. In addition, receiver operating characteristics (ROCs) curve provided a predictive ability in the training sets (AUC = 0.789, 95% confidence interval [CI] 0.789–0.808) and the validation cohorts (AUC = 0.796, 95% confidence interval [CI] 0.744–0.841).

**Conclusion:**

In our study, the nomogram accurately predicted risk factors of LM in patients with chondrosarcoma, which may guide surgeons and oncologists to optimize individual treatment and make a better clinical decisions.

**Trial registration:**

JOSR-D-20-02045, 29 Dec 2020.

## Background

Chondrosarcoma is the third most common primary malignant bone tumor after myeloma and osteosarcoma and the second most common bone malignant neoplasms accounting for 30% of all primary malignant bone tumors. At present, surgical resection is the mainstay therapeutic option for chondrosarcoma, while the radiotherapy is only applied to patients with unresectable lesions or inoperable extensive marginal resection and the chemotherapy only recommended for young patients with good tolerance. However, the effects of treatment are insignificant according to the recent studies [1, 2].

According to previous research, approximately 8 to 38% of the patients with chondrosarcoma developed distant metastasis and lung was the preferred site of metastasis [3–6]. Furthermore, the 10-year survival rate and the metastasis rate of chondrosarcoma patients with lung metastasis were 17 and 9.6%, respectively. The occurrence of LM had a strong predictor of poor prognosis [7, 8]. In addition, up to 13% of recurrent chondrosarcomas got a higher grade of malignancy than the original neoplasm [9]. Due to the effects of metastasis, complete removal of the tumor became extremely difficult. Therefore, it was imperative to identify the risk factors of chondrosarcoma patients with lung metastasis [10].

Chondrosarcoma is a rare tumor, accounting for 20% of primary malignant bone tumor, with an estimated incidence rate of 1 per 200,000. The lack of clinical research in chondrosarcoma is subjected to the low and sporadic incidence of chondrosarcoma. The Surveillance, Epidemiology, and End Results (SEER) database that sponsored by the National Cancer Institute records cancer incidence and survival data from 18 population-based cancer registries. The database includes approximately 27.8% of the U.S. population and is publicly available [11].

With the advantage of visualization and accurate prediction, nomogram has been widely applied to predict the risk factors of metastasis and oncological outcomes [12]. With Nomogram, clinicians can assess the risk of clinical events, offer individual treatment plans, optimize treatment regimens, and be more active at follow-up. The purpose of this study is to construct a nomogram to evaluate high-risk group of chondrosarcoma patients with LM due to the importance of LM in the prognosis of chondrosarcoma patients,.

## Materials and methods

### Data collection

Data were extracted from the SEER database, using the SEER * Stat software 8.3.6 version. And the third edition of the International Taxonomy of Oncology (ICDO-3), morphological code(9220) was used to identify chondrosarcoma. Data in this study consisted of patients diagnosed with chondrosarcoma from 2010 to 2016. The exclusion criteria were as follows: (1) patients with no positive pathology; (2) patients with unknown survival time; (3) not the first-occurrence tumor; (4) more than one primary tumor; (5) lung metastasis information was unknown. (6) incomplete information on regional lymph node metastasis.

Demographic and clinical variables (including age, gender, race, marriage, main location, single or multiple tumors, tumor size and lymphatic metastasis) were considered in this study. These data were determined by the variable “CS site-specific factor 6”. Patients were classified as married and unmarried (including single, divorced, separated, and widowed). Besides, patients with less than 20 tumor sites were classified as “other”.

### Construction, validation and clinical utility of a nomogram

To investigate risk factors for LM at the initial diagnosis of chondrosarcoma, we extracted the data of patients diagnosed after 2010 year from SEER database [13], then randomly divided patients into the training sets(*n* = 644) and validation cohorts (*n* = 280).

Then, the following variables were chosen for research: age, race, gender, marriage, main location, single or multiple tumors, tumor size, and lymphatic metastasis. All the variables with a significance level *P* < 0.05 in univariate logistic regression analysis were included in the multivariable logistic regression analysis. The nomogram was constructed based on the results of the univariate and multivariable logistics regression analysis. The calibration plot of clinical prediction model and receiver operating characteristic (ROC) curves were used to estimate the prediction performance of the nomogram. The higher the area under the curves (AUC) of ROC, the better the model was. In addition, the decision curve analysis (DCA) was used to evaluate the clinical utility of nomograms in decision-making. The DCA is a kind of chart that can show net benefits under a series of reasonable risk thresholds in practice. CIC was developed based on DCA to visual display the estimated number of high-risk patients for each risk threshold.

### Statistic analysis

Continuous variables were expressed as mean ± standard deviation (SD), and categorical variables were expressed as frequency (proportions). Hypothesis test was utilized to compare the difference between metastatic and non-metastatic group. T-test and Chi-square were applied to continuous variables and categorical variables respectively by IBM SPSS Statistics version 26.0(SPSS Inc., Chicago, Illinois, USA). R software version 3.6.2 (http://www.r-project.org) including multiple R packages (Including regplot, rms, rmda and pROC) was employed to draw graphics, such as Nomogram, Calibration plot, DCA graph, ROC curve and KM curve. The *P* < 0.05 was considered as statistically significant, and confidence intervals (CIs) were expressed as 95% confidence levels.

## Results

### Demographic baseline characteristics

A total of 944 patients were engaged in this study. The baseline information categorized as training group and validation group. There was no statistically significant difference between the training and validation groups (*P* > 0.05) (Table 1).

**Table 1:** Baseline characteristics of training group and validation group

	Level	Overall (*n* = 944)	Training group (*n =* 664)	Validation group (*n =* 280)	p
Age (mean (SD))	NA	55.09 (18.08)	54.60 (18.24)	56.23 (17.67)	0.206
Sex (%)	female	405 (42.9)	291 (43.8)	114 (40.7)	0.418
	male	539 (57.1)	373 (56.2)	166 (59.3)	
Race (%)	black	67 (7.1)	51 (7.7)	16 (5.7)	0.429
	other	58 (6.1)	43 (6.5)	15 (5.4)	
	white	819 (86.8)	570 (85.8)	249 (88.9)	
Primary.site (%)	Aix of bones	389 (41.2)	274 (41.3)	115 (41.1)	0.606
	Limb bones	514 (54.4)	364 (54.8)	150 (53.6)	
	Other	41 (4.3)	26 (3.9)	15 (5.4)	
Marital (%)	no	344 (36.4)	249 (37.5)	95 (33.9)	0.333
	yes	600 (63.6)	415 (62.5)	185 (66.1)	
Sequence.number (%)	more	188 (19.9)	133 (20.0)	55 (19.6)	0.963
	only one	756 (80.1)	531 (80.0)	225 (80.4)	
Tumor.size (mean (SD))	NA	79.20 (66.42)	79.94 (72.61)	77.45 (48.77)	0.598
Lymph (%)	No	915 (96.9)	646 (97.3)	269 (96.1)	0.536
	unknow	20 (2.1)	13 (2.0)	7 (2.5)	
	yes	9 (1.0)	5 (0.8)	4 (1.4)	

### Univariate and multivariable logistic regression results

Based on the univariate logistics regression analysis, we identified five significant prognostic factors including age, sex, marital, tumor size and lymph metastasis in the training set (*P* < 0.05) (Table 2). Then, applying the multivariable logistics regression analysis, we figured out those independent prognostic factors including four protective factors containing gender (female: odds ratio (OR) 0.435, 95%CI 0.212-0.891, *P* < 0.05), age (OR = 1.026, 1.005-1.048, *P <* 0.05), tumor size (OR = 1.003, 1.003-1.006, *P <* 0.05) and lymph metastasis (Yes:OR = 27.164, 6.267-117.741, *P* < 0.0001; Unknown:8.027, 2.643-24.379, *P <* 0.0001) (Table 2).

**Table 2:** Univariate and multivariable logistic regression results of chondrosarcoma patients with pulmonary metastasis

Variables	Univariate		Multivariable	
	OR (95% CI)	*p* value	OR (95% CI)	*p* value
Age (years)	1.038 (1.019-1.057)	< 0.0001	1.026(1.005-1.048)	< 0.05
Race				
White	Ref	Ref	/	/
Black	1.237 (0.429-3.567)	0.694	/	/
Other	0.696 (0.164-2.953)	0.623	/	/
Sex				
Male	Ref	Ref	Ref	Ref
Female	0.454 (0.232-0.887)	< 0.05	0.435(0.212-0.891)	< 0.05
Marital				
No	Ref	Ref	Ref	Ref
Yes	2.840(1.309-6.160)	< 0.01	2.151(0.212-5.207)	0.89
Primary site				
Limb bones	Ref	Ref	/	/
Aix of bones	0.539(0.278-1.044)	0.067	/	/
Other	0.820 (0.189-3.3558)	0.791	/	/
Sequence number				
Only	Ref	Ref	/	/
More	0.840(0.385-1.831)	0.661	/	/
Tumor size (mm)	1.004(1.001-1.007)	< 0.01	1.003(1.000-1.006)	< 0.05
Lymph				
No	Ref	Ref	Ref	Ref
Yes	31.429(8.087-122.415)	< 0.0001	27.164 (6.267-117.741)	< 0.0001
Unknown	10.776 (3.908-29.712)	< 0.0001	8.027(2.643-24.379)	< 0.0001

### Construction and validation of nomogram for chondrosarcoma patients with pulmonary metastasis

**Fig. 1:**
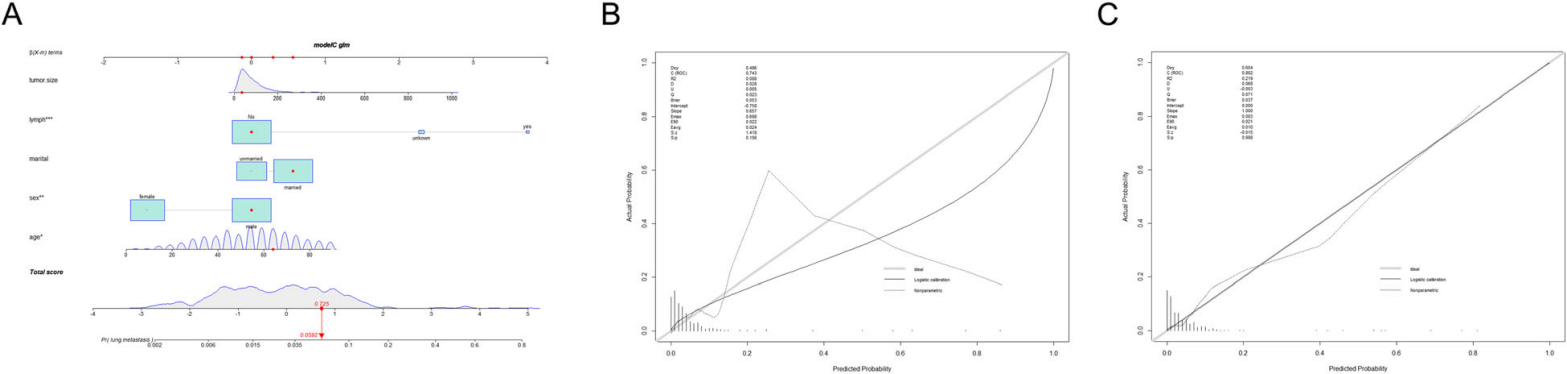
**a** Nomogram for the risk of pulmonary metastasis for chondrosarcoma patients. **b** and **c** are its training cohorts and the validation cohorts calibration diagrams respectively, which indicate good consistency

**Fig. 2:**
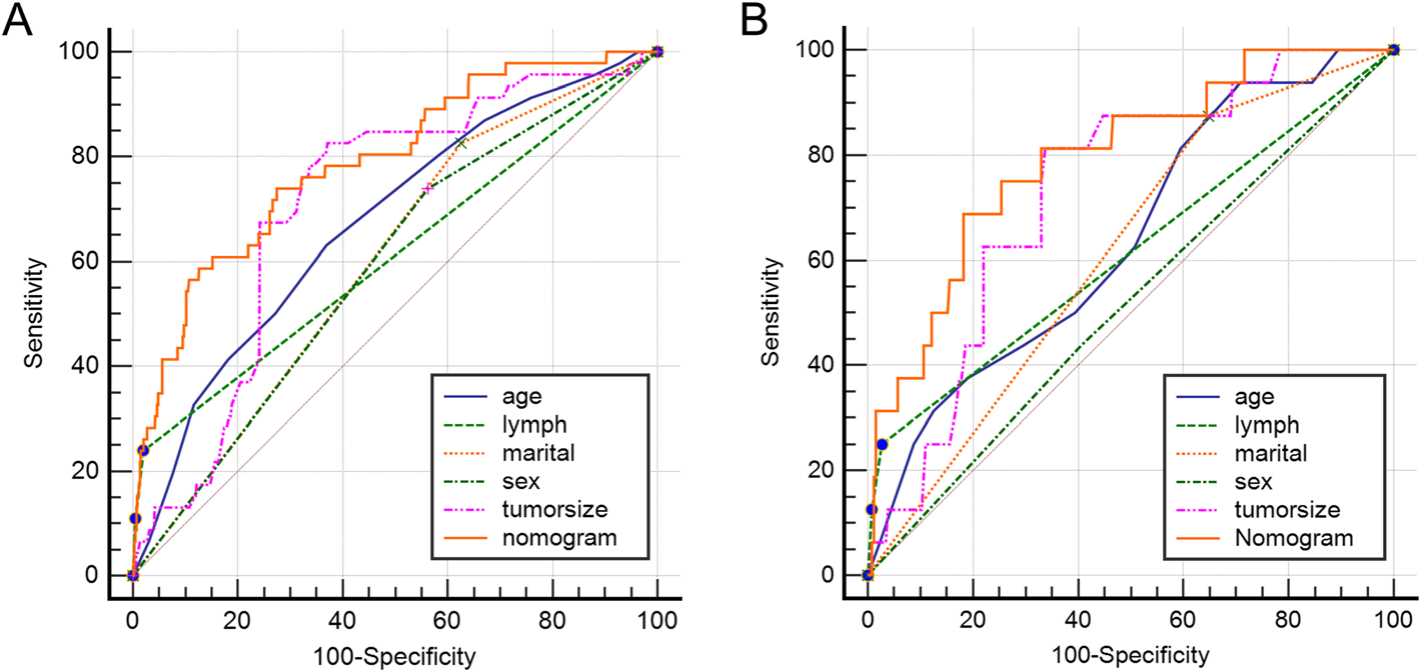
ROC of Nomogram for the pulmonary metastasis risk (**a** training group and **b** validation group)

The results of univariate and multivariable logistics regression were used to construct the Nomogram of LM (Fig. 1a). As shown in the Fig. 1, lymph contributed most to prognosis followed by tumor size, marital status, age and sex. The calibration chart of the Nomogram showed a good consistency in the training cohorts and the validation cohorts (Fig. 1b, c). The AUC values of Nomogram were 0.789 (95% CI 0.762-0.851) and 0.796 (95% CI 0.744-0.841) respectively in the training cohorts and the validation cohorts (Fig. 2a, b). Furthermore, the ROC curve displayed that the value of Nomogram was more important than other variables, including age (AUC = 0.674, 95%CI 0.644 to 0.704), lymph node metastasis (0.610, 0.578 to 0.641), and marital status (AUC = 0.600, 95%) CI 0.568 to 0.632), sex (AUC = 0.588, 95%CI 0.568 to 0.632) and tumor size (0.710, 95%CI 0.680 to 0.739) on training sets. The results of the validation cohorts indicated that the value of Nomogram was also higher than that of single factor as shown in Table 3.

**Table 3:** AUC values for the training and verification groups

Variable	Training group(*n* = 664)		Validation group(*n* = 280)	
	AUC	95%CI	AUC	95%CI
Age	0.674	0.644 to 0.704	0.640	0.581 to 0.697
Lymph	0.610	0.578 to 0.641	0.612	0.553 to 0.670
Marital	0.600	0.568 to 0.632	0.614	0.554 to 0.671
Sex	0.588	0.556 to 0.620	0.516	0.456 to 0.576
Tumor size	0.710	0.680 to 0.739	0.730	0.674 to 0.781
Nomogram	0.789	0.762 to 0.815	0.796	0.744 to 0.841

### Clinical applicability of the nomogram

**Fig. 3:**
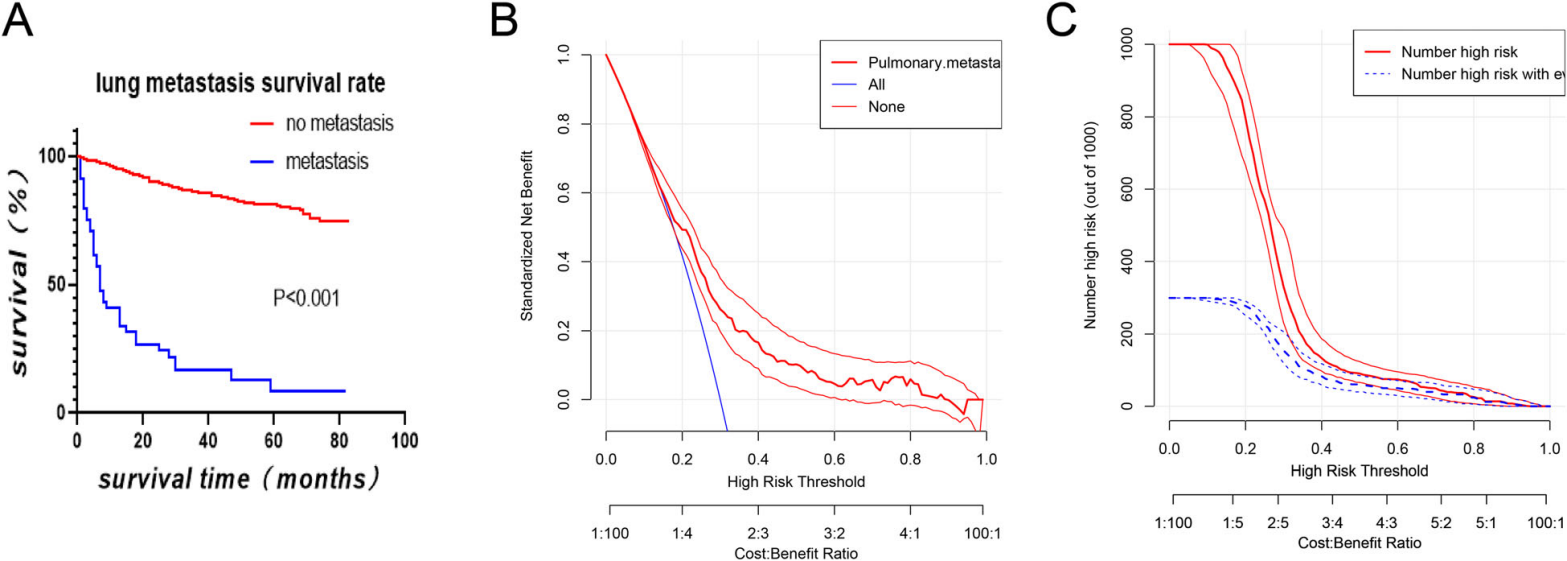
**a** The Kaplan-Meier survival analysis of lung metastasis in patients with chondrosarcoma. **b, c** Nomogram decision curve (DCA) and clinical implications (CIC) for the risk of the lung metastasis. The red curve (Number of high risk) indicates the Number of people classified as positive (high risk) by Nomogram for each threshold probability. The blue curve (number of high risk with outcome) represents the number of true positives under each threshold probability

The Kaplan-Meier survival curves of the overall survival (OS) of 944 patients were plotted (Fig. 3a). The results revealed that the survival significantly decreased in chondrosarcoma patients with LM comparing with the other group(*P* < 0.001). Moreover, the threshold about 0.1 to 0.8 had the maximum benefit range of the model as shown in the DCA curve (Fig. 3b). In addition, the clinical impact curve of the training cohorts revealed that within the most favorable threshold probability range, the number of predicted high-risk patients were always more than the actual patients with LM, accompanied by an acceptable cost-benefit ratio (Fig. 3c).

## Discussion

This study, we found that the following five independent risk factors including gender, age at the diagnosis, marital status, tumor size, and the lymph node involvement,were identified to be associated with LM applying univariate and multivariable logistic regression analysis on the data collected from the SEER database. All these factors were involved into the nomogram. The calibration plots showed the nomogram demonstrated good discrimination (Fig. 1b). Furthermore, as shown in the ROC curves (Table 3), the diagnostic efficiencies of the nomogram were better than any single predictors, which demonstrated the significance of a comprehensive predictive model. The DCA curve showed that the probability threshold for LM patients to obtain the maximum benefit is 0.1-0.8 (Fig. 3b). In addition, the CIC (Fig. 3c) shows that there is an acceptable cost-benefit ratio of the threshold range of. This nomogram could be used as a reliable graphic tool to help surgeons and oncologists distinguish and estimate the risk of lung metastasis and guide personalized treatment for chondrosarcoma patients.

According to a large population-based study, approximately 8% of patients with chondrosarcoma developed distant metastases [14]. The poor prognosis of patients with chondrosarcoma might be associated with the lung metastasis [15]. Therefore, we intended to identify risk factors of chondrosarcoma patients with lung metastasis [8, 16].

The visual nomogram is well known for its predictive accuracy and has made remarkable contributions to modern medical decision-making [17, 18]. Zhang et al. [19] and Song et al. [4, 20] constructed and validated nomograms to predict the overall survival (OS) and cancer-specific survival (CSS) of chondrosarcoma patients. However, it was an innovation that establishing Nomogram based on the SEER database to analyze the high risk of lung metastases in chondrosarcoma.

Generally speaking, regional lymph node metastasis developed quite rarely in chondrosarcoma, and the incidence was approximately 1.3% across all chondrosarcoma [21]. The low prevalence of lymph node involvement might be related to the scarce lymphatic vessels in normal bones, benign tumors and malignant neoplasms. In addition, the research indicated that lymphatic vessels existed in the connective tissue covering the periosteum, and lymphatic metastasis occurred only when the tumor broke through the periosteum and invaded adjacent connective tissue [22]. Patients with this clinicopathological behavior might be diagnosed with a more aggressive chondrosarcoma.

According to the results of logistic regression analysis, taking patients without lymph node metastasis as the baseline, the risk ratios of lung metastasis for patients with lymph node involvement (OR = 27.164) and patients with unknown lymph node metastasis (OR = 8.027) were 27.164 and 8.027 respectively (Table 2). Considering regional lymph node involvement can result in a strong adverse prognostic impact and relatively higher risk of lung metastasis, we recommend biopsy for suspicious patients. Studies verified that it was essential to determine the presence of lymph node metastasis, which corroborated our conclusion [23]. Further research might be focused on applying this association to improve patient long-term survival.

The tumor size was associated with the risk for developing lung metastasis according to the statistical results acquired from univariate and multivariable logistic regression. In particular, the calculated OR value was 1.003, which represented that for each 1 mm increase in tumor size, the risk of LM increased by 1.003 times. Relevant study revealed that larger tumors represent a longer period of time for tumors growth, increasing the likelihood of metastasis [24]. Consistent with our findings, recent studies reported that tumor size was a significant independent predictor of LM and mortality [16, 25, 26], especially when the tumor size exceeding 10 cm [27].

Besides, the older patients with chondrosarcoma had a higher tendency developed with LM and had more negative prognosis conforming to the result of logistic regression. Previous studies identified the same results [28]. The increase in the risk of lung metastasis was 1.026 for each additional 1-year increase in the age of diagnosis with a reference of 60 years (Table 2). Moreover, a retrospective study found that age over 60 was an independent risk factor for LM and revealed the reason of poor prognosis in elderly patients [26]. The main reason why chondrosarcoma is not easily diagnosed and treated early is the late production of obvious symptoms such as swelling, pressure, and pain, especially for older patients, resulting in many patients presenting to the clinic with tumors that have progressed to a more advanced stage, leading to a higher risk of LM and affecting patient prognosis. Therefore, clinicians need to be more alert to the presence of LM in patients with advanced chondrosarcoma.

In addition, the result of logistic regression revealed males had a higher risk than females developing LM. As shown in OR value, the ratio of males and females with LM was 1:0.4. Therefore, we reasonably speculate that males had a more adverse survival expectancy. The standpoint was supported by several studies that indicated sex was an independent risk factor affecting the long-term prognosis of chondrosarcoma patients [24, 29, 30]. One possible reason for the worse prognosis of male patients compared to female patients is that male patients possess a higher risk of LM. Researchers also need to consider that male patients have a higher proportion of adverse habits, such as smoking and alcohol abuse, as well as poorer medical vigilance. These habits may cause men to come to the doctor at a worse level of cancer progression compared to women. This also leads to a higher risk of LM in males.

In addition, it was worth noting that this study considered the marital status as a risk factor on LM. We determined that comparing to unmarried patients, married chondrosarcoma patients with LM had significant survival benefits, which revealed that marital status was associated with a better prognosis [31, 32]. Favorable financial conditions and emotional support from spouses contributed to cultivate the married patients’ treatment adherence and regular follow-ups. Meanwhile, cancer-caused survival was also found to be relatively poor in widowed chondrosarcoma patients. This may be related to the greater psychological stress suffered by widowed patients as well as the greater stress associated with adjusting to a new social role. Unfortunately, the detailed economic status of patients was unavailable in the SEER database, so the impact of finance on LM cannot be further studied.

Finally, several limitations in our study need to be treated with caution. First, the information about asymptomatic LM patients and metastasis during follow-up is not recorded in the SEER database. Second, our retrospective study inevitably leads to a bias by the lack of lack systematic and prospective data. In our single-center study, the method of dividing patients into training and validation cohorts cannot be completely equivalent to external validation at other institutions, which might appear an overfitting LM nomogram.

## Conclusion

In summary, we constructed a novel nomogram to predict risk factors for chondrosarcoma patients developing LM, including sex, age, tumor size, marital status and tumor size based on epidemiological characteristics obtained from the SEER database. By combining DCA curve, clinical impact curve and internal validation, our nomogram provided an accurate assessment for individualized risk of LM which guided clinicians to optimize personalized treatment and make superior clinical-related decisions.

## Data Availability

The dataset supporting the conclusions of this paper can be obtained from the SEER database.

## Abbreviations

LM: Lung Metastasis
SEER: The Surveillance, Epidemiology, and End Results database
ROC: Receiver Operating Characteristic
DCA: Decision Curve Analysis
CIC: Clinical Impact Curve
CI: Confidence Interval
AUC: Area Under the Curve
ICD: International Classification of Diseases
SD: Standard deviation
KM curves: Kaplan Meier curves
OR: Odds ratio
